# Anthelmintic efficacy and safety of alkaloid-rich fractions of *Nicotiana tabacum* against benzimidazole-resistant *Haemonchus contortus* in goats

**DOI:** 10.14202/vetworld.2025.3420-3432

**Published:** 2025-11-23

**Authors:** Muhammad Sheraz Yasin, Wasim Shehzad, Kamran Ashraf, Rahat Naseer, Khalid Hussain

**Affiliations:** 1 Institute of Biochemistry and Biotechnology, University of Veterinary and Animal Sciences, Lahore, Pakistan; 2 Department of Parasitology, University of Veterinary and Animal Sciences, Lahore, Pakistan; 3 Department of Pharmacy, Faculty of Pharmacy, The University of Lahore, Lahore, Pakistan

**Keywords:** alkaloids, anthelmintic resistance, benzimidazoles, goats, *Haemonchus contortus*, *Nicotiana tabacum*, One Health, Sustainable development goals 12 –, Responsible consumption and production, Sustainable development goals 15 –, Life on land, Sustainable development goals 2 –, Zero Hunger, Sustainable development goals 3 –, Good health and well-being, sustainable livestock

## Abstract

**Background and Aim:**

*Haemonchus contortus* is a highly pathogenic blood-feeding nematode of small ruminants, responsible for severe anemia, production losses, and mortality. Excessive use of synthetic anthelmintics, especially benzimidazoles, has led to widespread drug resistance, prompting a need for alternative therapeutics. *Nicotiana tabacum* (tobacco) contains bioactive alkaloids such as nicotine, which target nematode acetylcholine receptors and may provide sustainable parasite control. This study aimed to isolate, characterize, and evaluate the *in vitro* and *in vivo* anthelmintic efficacy and safety of alkaloid-rich fractions of *N. tabacum* against benzimidazole-resistant *H. contortus* in goats, in line with the One Health approach and the United Nations Sustainable Development Goals (SDGs 2, 3, 12, and 15).

**Materials and Methods:**

Leaves of *N. tabacum* were authenticated and subjected to sequential solvent extraction. Alkaloid fractions were confirmed by TLC and HPLC. *In vitro* assays, including adult motility and egg hatch tests, were conducted at concentrations of 1–5 mg/mL against resistant *H. contortus* isolates. The most active fraction (ethyl acetate) was administered orally in naturally infected Beetal goats (n = 25) at low (0.8 mg/kg), medium (1.2 mg/kg), and high (1.6 mg/kg) doses for 14 days, alongside negative (saline) and positive (oxfendazole 4.5 mg/kg) controls. Fecal egg counts, hematology, and liver enzyme levels were analyzed to determine efficacy and safety.

**Results:**

The ethyl acetate fraction exhibited complete (100%) adult worm mortality at 3–5 mg/mL and total egg-hatch inhibition at 4–5 mg/mL (p < 0.05). The LD_50_ for adult worm mortality was 0.323 mg/mL. *In vivo*, the high-dose group (1.6 mg/kg) achieved a 76.2% fecal-egg-count reduction, exceeding oxfendazole (69.7%). No significant changes in alanine aminotransferase or aspartate aminotransferase were observed (p > 0.05), confirming hepatic safety, while serum proteins and red-blood-cell indices improved significantly (p < 0.05).

**Conclusion:**

Purified alkaloid fractions of *N. tabacum*, particularly the ethyl acetate extract containing nicotine, demonstrated strong, dose-dependent anthelmintic activity, and safety against benzimidazole-resistant *H. contortus*. These findings support *N. tabacum* as a sustainable, plant-based alternative to synthetic anthelmintics. The work advances the One Health framework and contributes directly to SDG 2 (Zero Hunger), SDG 3 (Good Health and Well-being), SDG 12 (Responsible Consumption and Production), and SDG 15 (Life on Land).

## INTRODUCTION

*Haemonchus contortus* is a hematophagous nematode that inhabits the abomasum of small ruminants [1]. Due to its characteristic spiraled appearance, it is commonly referred to as the barber’s pole worm [2]. Infection with *H. contortus* causes substantial morbidity and mortality in small ruminants, resulting in significant production losses [3]. The parasite interferes with growth and productivity by inducing diarrhea, inappetence, anemia, and, in severe cases, death [4]. The global prevalence of *H. contortus* in small ruminants ranges from 52.7% to 83%, depending on region and climatic conditions [5]. The incidence of hemonchosis is highly seasonal, peaking during hot and humid month (38.70% in July) and declining during cooler month (11.11% in December) [6].

In Pakistan, the livestock sector plays a pivotal role in the national economy, contributing approximately 60.85% to the agricultural value and 14.63% to the national GDP [7]. However, the susceptibility of small ruminants to *H. contortus* infection results in substantial financial losses to the livestock industry worldwide [8]. Although the exact figures vary depending on regional production systems and infection intensity, global estimates suggest annual losses amounting to hundreds of millions of dollars [9, 10]. In Pakistan alone, economic losses due to hemonchosis are estimated to exceed 8,800 million Pakistani rupees annually, attributed to condemned abomasa and a 29% decline in milk yield. The total annual financial impact, including mortality in young animals, is approximately 142,902 million Pakistani rupees [11].

Conventional control of *H. contortus* relies predominantly on synthetic chemotherapeutics [12]. Commonly used anthelmintics include piperazines, benzimidazoles, morantel, pyrantel, levamisole, tribendimidine, avermectins, and milbemycins [13–17]. However, the indiscriminate and frequent use of these drugs has led to the emergence of resistance among *H. contortus* populations worldwide, including in Pakistan [18, 19]. Previous studies Iqbal *et al*. [20], Hamad *et al*. [21], Chaudhry *et al*. [22], and Sangster *et al*. [23] have documented benzimidazole resistance in *H. contortus* isolates from Pakistan. Genetic investigations have identified mutations at the β-tubulin gene locus as the principal mechanism conferring benzimidazole resistance [24]. Consequently, researchers are increasingly exploring plant-derived alternatives to mitigate drug resistance and provide sustainable parasite control [25].

Medicinal plants represent promising candidates for combating parasitic infections in livestock and other species [26–31]. A previous *in vitro* study by Abubakar *et al*. [26] has demonstrated that several plant extracts exhibit inhibitory effects on the developmental stages of *H. contortus*. In Pakistan, many livestock farmers traditionally rely on herbal remedies for managing helminthiasis [32]. Previous studies Akhtar *et al*. [33], Carvalho *et al*. [34], and Ghisi *et al*. [35] across the world have evaluated the anthelmintic activity of plant species against nematode infections in animals and humans. Plants such as *Nicotiana tabacum*, *Chenopodium ambrosioides*, *Lawsonia inermis*, *Jatropha curcas*, *Annona muricata*, *Iris kashmiriana*, *Cissus quadrangularis*, *Schinus molle*, *Murraya koenigii*, and *Azadirachta indica* have demonstrated anthelmintic efficacy in both *in vitro* and *in vivo* models [36–39]. Similarly, *Pithecellobium dulce* extracts have been reported to inhibit larval development of *H. contortus* [40]. A crude ethanolic extract of *N. tabacum* at 100 mg/mL caused 91% larval mortality within 10 min and achieved complete clearance of larvae from infected sheep livers within 20–60 min [41]. Furthermore, methanolic extracts of *N. tabacum* have shown significant *in vivo* efficacy against *H. placei* in cattle, reinforcing the potential of tobacco-derived compounds as effective anthelmintics [42].

Although numerous studies have evaluated the anthelmintic potential of medicinal plants against *H. contortus*, the majority have relied on crude extracts rather than purified bioactive compounds. Crude plant extracts often contain complex mixtures of phytochemicals whose individual effects, potency, and toxicity remain poorly characterized. This compositional variability, arising from differences in plant genotype, soil composition, climate, and extraction methods, leads to inconsistent pharmacological responses and challenges in reproducibility and standardization. Specifically, *N. tabacum* has demonstrated strong *in vitro* efficacy against various nematodes, including *H. contortus* and *H. placei*; however, yet prior investigations have not isolated or quantified the active alkaloid fractions responsible for these effects. Moreover, most existing work is limited to *in vitro* laboratory assays, with scarce data confirming the *in vivo* efficacy, dose–response relationships, and safety profiles of purified alkaloids in naturally infected animals. There is also a limited understanding of whether these bioactive fractions can be safely administered to small ruminants without adversely affecting hematological or hepatic parameters. Hence, a scientifically controlled study investigating both *in vitro* and *in vivo* effects of purified *N. tabacum* alkaloids against benzimidazole-resistant *H. contortus* is critically needed to bridge this knowledge gap and support the development of plant-based alternatives to synthetic anthelmintics.

This study was designed to isolate, purify, and characterize the alkaloid-rich fractions of *N. tabacum* leaves and to evaluate their anthelmintic efficacy and safety against benzimidazole-resistant *H. contortus* in goats. The specific objectives were as follows:

1. To extract and purify alkaloid fractions from *N. tabacum* using solvent fractionation, TLC, and HPLC confirmation of nicotine content
2. To assess the *in vitro* anthelmintic activity of different fractions at variable concentrations (1–5 mg/mL) through adult motility and egg hatch inhibition assays
3. To determine the *in vivo* efficacy of the most active fraction in goats naturally infected with resistant *H. contortus* by monitoring reductions in fecal egg counts (eggs per gram [EPG])
4. To evaluate the hematological, biochemical, and liver function profiles of treated animals to establish the safety of the purified alkaloid fractions.

By integrating phytochemical analysis, *in vitro* bioassays, and *in vivo* trials, this research aimed to identify an effective and safe plant-derived alternative to synthetic anthelmintics and contribute to sustainable parasite management within the One Health framework.

## MATERIALS AND METHODS

### Ethical approval

All experimental procedures involving animals were carried out in strict accordance with the ethical principles and guidelines for animal experimentation approved by the Institutional Ethical Review Committee of the University of Veterinary and Animal Sciences, Lahore, Pakistan (Approval No. DR/357, dated July 25, 2023). The study design complied with the Animal Welfare Act (2006, Pakistan), the Animal Research: Reporting of *In Vivo* Experiments (ARRIVE) 2.0 guidelines, and the recommendations of the World Organization for Animal Health (WOAH) for the humane care and use of animals in research.

All goats were monitored daily by a registered veterinarian to ensure animal health and well-being throughout the experimental period. The number of animals used was kept to the minimum necessary to achieve reliable scientific results. No invasive or painful procedures were performed beyond standard handling and sample collection. All animals were returned to farm management conditions after the completion of the study.

### Study period and location

This study was conducted between January 2021 and November 2023 at Postgraduate Laboratory, Institute of Biochemistry and Biotechnology, University of Veterinary and Animal Sciences, Lahore, Pakistan.

### Plant material and authentication

*N. tabacum* (tobacco), a Rabi crop, was selected for evaluation. The leaves were purchased from a local market in Lahore, Pakistan, and authenticated by the Department of Botany, Government College University, Lahore. A voucher specimen (Gc. Herb. Bot. 3988) was deposited for reference.

### Preparation and fractionation of alkaloid-rich extracts

#### Preparation of crude extract

The collected leaves were air-dried at room temperature (25°C) and cleaned of dust and fungal contaminants using distilled water. The dried leaves were ground into fine powder (mesh size 0.1 mm) using an electric grinder and stored in airtight cellophane bags. The acidic aqueous extract was prepared according to the method of Phukan *et al*. [43] with minor modifications. Briefly, acidic distilled water (pH 4) was added to the powdered leaves and heated at 40°C for 1 h to obtain the crude extract.

#### Solvent fractionation

The crude extract was subjected to solvent–solvent fractionation at a ratio of 50 mg/50 mL using chloroform, n-hexane, and distilled water. The resulting fractions were analyzed by thin-layer chromatography (TLC) and high-performance liquid chromatography (HPLC) to confirm the presence of alkaloids [44].

#### Purification of alkaloid fractions

The chloroform fraction, showed the highest alkaloid content, was further purified by column chromatography on silica gel. Petroleum ether, ethanol–water (7:3), ethyl acetate, and distilled water were employed as mobile phases to obtain different sub-fractions. These purified fractions were analyzed using HPLC before their application in *in vitro* assays.

### *In vitro* assays against benzimidazole-resistant *H. contortus*

#### Collection and culturing of parasite eggs

Fecal samples containing *H. contortus* eggs were collected from naturally infected goats maintained at the Small Ruminants Training and Research Center, University of Veterinary and Animal Sciences, Ravi Campus, Pattoki, Pakistan. The eggs were cultured and maintained according to established methods [45–47].

To minimize the risk of toxicity, five concentrations (1–5 mg/mL) of the purified alkaloid fractions were selected for *in vitro* assays based on previously reported protocols [48], with minor modifications. Specifically, the experiments were conducted in 96-well ELISA plates, maintaining a total reaction volume of 350 μL per well.

#### Adult motility test (AMT)

The adult motility test (AMT) was conducted according to the method described by García-Hernández *et al*. [49], with minor modifications, to evaluate the efficacy of the purified fractions against oxfendazole-resistant *H. contortus* adults. Specifically, the assay was performed in 96-well ELISA plates with a total reaction volume of 350 μL per well. Ten live adult worms were suspended in phosphate-buffered saline (PBS) in each well of a 24-well flat-bottom plate. The purified fractions (petroleum ether, ethanol–water (7:3), ethyl acetate, and distilled water) were tested at concentrations of 1.0–5.0 mg/mL in triplicate. PBS served as the negative control, while oxfendazole (25 μg/mL; Oxfenda, GlaxoSmithKline, Pakistan) in PBS served as the positive control.

All experiments were conducted at room temperature (~25°C). The worms were observed for motility, paralysis, and mortality at 0, 2, 4, 6, 8, 10, and 24 h. Non-motile worms were transferred to lukewarm water to confirm death. Worm motility and mortality were recorded as described by Abubakar *et al*. [26].

#### Egg hatch assay (EHA)

The EHA was performed to evaluate the inhibitory effect of the purified alkaloid fractions on egg hatching, following the method of Coles *et al*. [50] with minor modifications [51] including, change in concentrations of purified fractions, further, the positive control was treated with different synthetic anthelmintic.

Each fraction (petroleum ether, ethanol–water (7:3), ethyl acetate, and distilled water) was tested at five concentrations (1.0–5.0 mg/mL), each in triplicate. Approximately 25 eggs per well were counted using the McMaster technique, the gold standard for parasitic egg quantification, capable of detecting ≥25 eggs/g of feces [52].

The positive control group received 0.025 mg/mL oxfendazole (dissolved in 0.3% dimethyl sulfoxide), while the negative control received 350 μL PBS. The inhibition percentage of egg hatching was recorded after 1 week of incubation.

### *In vivo* anthelmintic evaluation

#### Experimental animals and treatment design

Twenty-five Beetal goats (13–15 months old; 35–40 kg) naturally infected with benzimidazole-resistant *H. contortus* were selected for the study based on a sample size determined through power analysis using G*Power version 3.1 (effect size = 0.40, α = 0.05, power = 0.80). The goats were maintained under semi-intensive housing, grazed on *Sesbania sesban* (Jantar) for 8 h daily, and provided ad libitum access to water.

The animals were randomized into five groups (n = 5/group) by lottery method, as shown in Table 1.

**Table 1:** *In vivo* study design, representing the different doses administered to the animals (n = 5) in each group.

Group 1	Group 2	Group 3	Group 4	Group 5
Control Negative Normal Saline	Control Positive Oxfendazole 4.5 mg/kg	Low dose of 0.81 mg/kg (Most effective fraction)	*N. tabacum* medium dose 1.2 mg/kg (Most effective fraction)	High dose of *N. tabacum* 1.6 mg/kg (Most effective fraction)

*N. tabacum* = *Nicotiana tabacum.*

- Group I: Control negative (normal saline)
- Group II: Control positive (oxfendazole, 4.5 mg/kg)
- Group III: Low dose (purified fraction, 0.8 mg/kg)
- Group IV: Medium dose (purified fraction, 1.2 mg/kg)
- Group V: High dose (purified fraction, 1.6 mg/kg)

Dose estimation was based on *in vitro* findings, LD_50_ values obtained from Probit analysis, and AMT results. Each goat received a single oral treatment, and the trial was conducted for 14 days.

#### Sample collection and outcome measures

Fecal samples were collected directly from the rectum of each goat on day 0 (pre-treatment) and day 14 (post-treatment) to determine EPG of feces using standard parasitological procedures [50].

Blood samples were drawn from the jugular vein on days 0 and 14 to evaluate:

- Liver function tests: Alanine aminotransferase (ALT) and aspartate aminotransferase (AST)
- Serum biochemistry: Total protein, serum albumin, serum globulin, and albumin/globulin ratio
- Hematology: Complete blood count (CBC)

Biochemical analyses were conducted using a Microlab 300 semi-automatic chemistry analyzer (ELITech Group, Netherlands) with Erba Lachema diagnostic kits (Erba Group, Czech Republic), while hematological parameters were measured using a URIT 3000VET hematology analyzer (URIT Medical Electronic Co., Ltd., China).

### Statistical analysis

Data on egg hatch inhibition, adult motility, fecal egg count reduction (EPG), liver enzymes, serum biochemistry, and hematological parameters were analyzed using one-way Analysis of Variance followed by Duncan’s multiple range test in IBM Statistical Package for the Social Sciences version 20.0 (IBM Corp., NY, USA). LD_50_ values were determined by Probit analysis. Sample size adequacy was confirmed through power calculation. Statistical significance was set at p < 0.05.

## RESULTS

### Yield and phytochemical characterization of plant extract

A total of 700 g of crude extract was obtained from 10 kg of *N. tabacum* leaves. Preliminary phytochemical screening using TLC confirmed the presence of alkaloids. HPLC analysis of the purified fractions further confirmed the presence of nicotine, quantified as follows: petroleum ether (0.10 mg/kg), ethanol–water (7:3; 2.30 mg/kg), ethyl acetate (7.12 mg/kg), and distilled water (0.18 mg/kg). The ethyl acetate fraction exhibited the highest alkaloid concentration and was selected for subsequent *in vitro* and *in vivo* evaluations.

### *In vitro* anthelmintic efficacy

#### AMT

**Figure 1:**
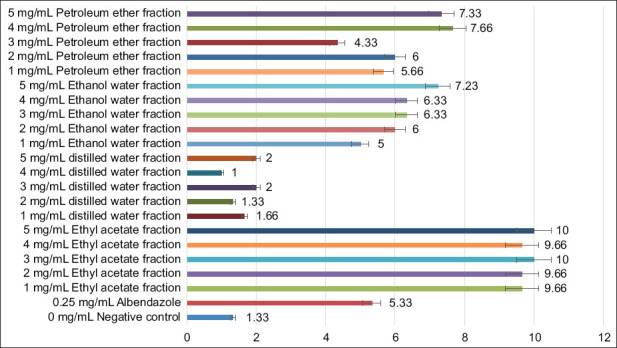
*In vitro* adult motility test to assess the effect of various concentrations of purified fractions of *Nicotiana tabacum* leaf extracts. The X-axis represents the mortality rate of worms, while the Y-axis represents various concentrations of purified fractions.

Among all purified fractions, the ethyl acetate fraction showed the strongest anthelmintic activity against adult *H. contortus* worms. Complete (100%) mortality was achieved at 3 and 5 mg/mL concentrations, with mean mortality values of 10.00 ± 0.00 and 10.00 ± 0.00, respectively (p < 0.05; Figure 1).

Probit analysis indicated an LD_50_ value of 0.323 mg/mL, while LD_90_ and LD_98_ values were 1.643 and 2.439 mg/mL, respectively, after 5 h of exposure. These results demonstrated potent, dose-dependent larvicidal activity of the ethyl acetate fraction.

#### EHA

**Figure 2:**
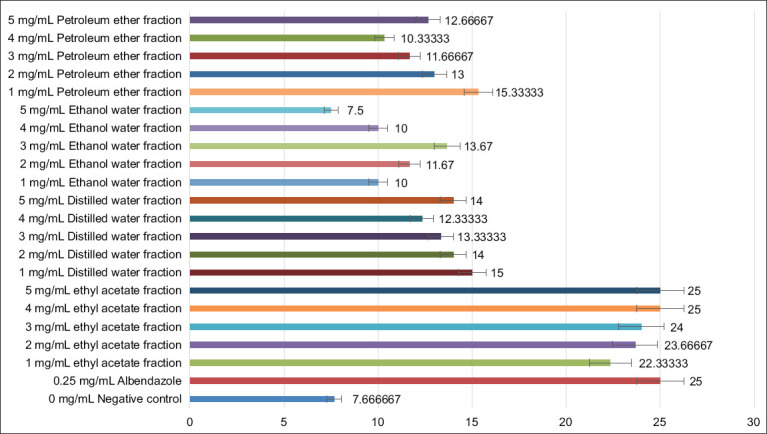
*In vitro* egg hatch assay for assessing the effect of various concentrations of purified fractions of *Nicotiana tabacum* leaf extracts. The X-axis represents egg hatch inhibition, and the Y-axis represents various concentrations of purified fractions.

The ethyl acetate fraction also showed the highest egg hatch inhibition among all tested fractions. Concentrations of 4 and 5 mg/mL inhibited egg hatching by 100% (mean values: 25.00 ± 0.00 for both; p < 0.05; Figure 2).

The petroleum ether, ethanol–water, and distilled water fractions exhibited comparatively lower inhibition rates. These findings confirm the ethyl acetate fraction as the most active alkaloid-rich extract for subsequent *in vivo* assessment.

### *In vivo* evaluation in goats

#### Fecal egg count reduction (EPG)

**Figure 3:**
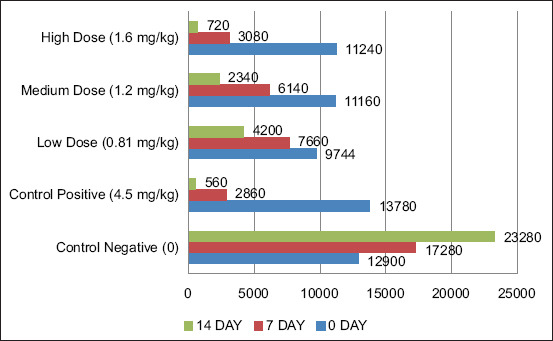
Effect of various concentrations of purified ethyl acetate fraction of *Nicotiana tabacum* leaf extracts on the number of eggs per gram in the feces of small ruminants. The X-axis represents the sum of eggs per gram, whereas the Y-axis represents the different doses of the tested compounds.

*In vivo* trials revealed a significant dose-dependent reduction in fecal egg counts across all treatment groups (p < 0.05; Table 2). The high-dose group (1.6 mg/kg) of the ethyl acetate fraction demonstrated the highest efficacy, achieving a 76.2% reduction in EPG by day 14 post-treatment (Figure 3).

**Table 2:** Mean EPG values for pre- and post-treatment with purified fraction in *in vivo* trials.

Trial duration	Groups					p-value (0.05)

	Control negative 0 mg/kg	Control positive oxfendazole 4.5 mg/kg	Low dose purified fraction 0.81 mg/kg	Medium-dose purified fraction 1.2 mg/kg	High-dose purified fraction 1.6 mg/kg	
Mean*EPG						
Day 0 of pretreatment	12900.0 ± 2098.5	13780.0 ± 2638.2	9744.0 ± 1235.2	11160.0 ± 1195.6	11240.0 ± 656.2	0.508
Day 7 post-treatment	17280.0 ± 2357.6	2860.0 ± 721.5	7660.0 ± 806.5	6140.0 ± 824.3	3080.0 ± 297.3	0.000
Day 14 post-treatment	23280.0^a^** ± 2217.7	560.0^c^** ± 153.6	4200.0^b^** ± 816.7	2340.0^bc^ ** ± 473.9	720.0^c^** ± 37.4	0.000

* Means ± standard error of the mean.

** Superscripts a, b, and c represent significant differences among the treatment groups. EPG = Egg per gram.

**Figure 4:**
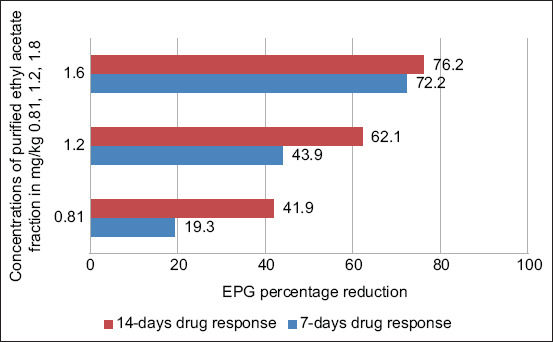
Dose response of 0.81, 1.2, and 1.8 mg/kg of ethyl acetate purified fraction of *Nicotiana tabacum* leaf extracts on eggs per gram in feces of small ruminants in *in vivo* trials. The X-axis represents the eggs per gram reduction percentage, while the Y-axis represents the dose concentrations.

This reduction was comparable to the positive control group treated with oxfendazole (69.7%). The dose–response curve (Figure 4) confirmed a clear relationship between increasing dose and parasitic load reduction, validating the *in vitro* results.

#### Liver function tests (ALT and AST)

No significant differences (p > 0.05) were observed in ALT or AST levels between the treated groups and the oxfendazole control on day 0 and day 14 (Table 3).

**Table 3:** Mean ALT and AST values before and after purified fraction treatment *in vivo* trial.

Group	Concentration	Mean*ALT U/L		Mean* AST U/L	
	
		Day 0 of pretreatment	Day 14 post-treatment	Day 0 of pretreatment	Day 14 post-treatment
Control negative	0 mg/kg	23.200 ± 1.984	18.400 ± 1.363	99.600 ± 5.801	93.800 ± 3.624
Control positive oxfendazole	4.5 mg/kg	22.000 ± 1.516	19.000 ± 0.707	104.800 ± 9.531	88.600 ± 7.743
Low-dose purified fraction	0.81 mg/kg	19.600 ± 0.979	18.400 ± 0.812	93.600 ± 10.385	103.000 ± 4.795
Medium-dose purified fraction	1.2 mg/kg	16.000 ± 1.788	16.200 ± 2.107	87.600 ± 2.925	100.200 ± 7.109
High-dose purified fraction	1.6 mg/kg	22.200 ± 3.261	19.600 ± 2.227	107.200 ± 5.276	88.800 ± 10.106

* Means ± standard error of the mean. ALT = Alanine aminotransferase, AST = Aspartate aminotransferase.

These findings indicate that the purified *N. tabacum* fractions, including the high dose (1.6 mg/kg), were non-hepatotoxic and safe for administration in small ruminants.

#### Serum protein profile

Serum biochemical analysis revealed a significant improvement (p < 0.05) in total protein, albumin, globulin, and albumin/globulin ratio across all treated groups by day 14 (Table 4).

**Table 4:** Mean total protein, serum albumin, serum globulin, and serum albumin globulin ratio before and after purified fraction treatment *in vivo* trial.

Group	Concentration	Mean* total protein (g/dL)		Mean* serum albumin level g/dL	
	
		Day 0	Day 14	Day 0	Day 14
Control negative	0 mg/kg	5.200 ± 0.173	5.840 ± 0.136	1.460 ± 0.269	1.720 ± 0.190**
Control positive Oxfendazole	4.5 mg/kg	5.420 ± 0.188	6.800 ± 0.118**	1.260 ± 0.087	3.160 ± 0.097**
Low dose purified fraction	0.81 mg/kg	5.733 ± 0.120	7.100 ± 0.114**	1.800 ± 0.200	3.560 ± 0.193**
Medium-dose purified fraction	1.2 mg/kg	5.620 ± 0.073	7.420 ± 0.135**	1.880 ± 0.096	3.580 ± 0.124**
High-dose purified fraction	1.6 mg/kg	5.300 ± 0.137	6.940 ± 0.196**	1.920 ± 0.330	3.320 ± 0.115**

**Group**	**Concentration**	Mean*serum globulin (g/dL)		Mean* albumin globulin ratio	
	
		**Day 0**	**Day 14**	**Day 0**	**Day 14**

Control negative	0 mg/kg	3.740 ± 0.290	4.120 ± 0.281	0.420 ± 0.131	0.440 ± 0.060**
Control positive Oxfendazole	4.5 mg/kg	4.160 ± 0.199	3.640 ± 0.172	0.300 ± 0.031	0.880 ± 0.058**
Low dose purified fraction	0.81 mg/kg	4.120 ± 0.201	3.540 ± 0.166	0.260 ± 0.112	1.020 ± 0.101**
Medium-dose purified fraction	1.2 mg/kg	3.740 ± 0.102	3.840 ± 0.120	0.520 ± 0.037	0.920 ± 0.048**
High-dose purified fraction	1.6 mg/kg	3.380 ± 0.330	3.620 ± 0.096	0.620 ± 0.174	0.920 ± 0.020**

* Means ± standard error of the mean.

** p < 0.05.

The medium-dose group (1.2 mg/kg) showed the highest increase in total protein levels (7.420 ± 0.135 g/dL), surpassing that of the positive control (6.800 ± 0.118 g/dL). Similarly, serum albumin, globulin, and A/G ratios improved markedly, suggesting restoration of physiological balance and absence of adverse metabolic effects following treatment.

#### Hematological parameters (CBC)

Significant improvements (p < 0.05) were recorded in hematological parameters, including hemoglobin concentration, red blood cell count, hematocrit percentage, mean corpuscular volume, and differential leukocyte counts on day 14 post-treatment (Table 5).

**Table 5:** Effect of different concentrations of ethyl acetate fractions on complete blood count in *in vivo* trials.

Group	Mean* hemoglobin g/dL		Mean* red blood cell count 10^6^/mm^3^		Mean*hematocrit percentage (%)	
		
	Day 0	Day 14	Day 0	Day 14	Day 0	Day 14
Control negative 0 mg/kg	7.640 ± 0.440	5.560 ± 0.250**	7.780 ± 0.409	5.320 ± 0.159**	23.560 ± 1.710	16.840 ± 0.494**
Control positive Oxfendazole 4.5 mg/kg	6.480 ± 0.753	8.133 ± 0.176**	7.350 ± 0.543	7.520 ± 0.239**	20.280 ± 2.236	24.200 ± 0.611**
Low dose purified fraction 0.81 mg/kg	7.560 ± 0.294	8.420 ± 0.662**	7.475 ± 0.249	7.620 ± 0.595**	23.340 ± 0.745	24.200 ± 1.896**
Medium- dose purified fraction 1.2 mg/kg	8.420 ± 0.424	9.520 ± 0.581**	7.833 ± 0.218	8.900 ± 0.187**	27.540 ± 1.305	28.066 ± 0.983**
High-dose purified fraction 1.6 mg/kg	7.040 ± 0.875	8.660 ± 0.201**	7.280 ± 0.848	8.540 ± 0.163**	22.100 ± 2.638	26.040 ± 0.624**

**Group**	Mean* corpuscular volume fL		Mean* neutrophil %		Mean* lymphocytes (%)	
		
	**Day 0**	**Day 14**	**Day 0**	**Day 14**	**Day 0**	**Day 14**

Control negative 0 mg/kg	30.300 ± 1.489	31.800 ± 1.599	37.500 ± 0.500	40.900 ± 0.979**	57.600 ± 0.927	53.800 ± 0.860**
Control positive Oxfendazole 4.5 mg/kg	30.025 ± 0.160	30.666 ± 0.976	41.400 ± 2.039	46.400 ± 1.964**	55.600 ± 2.420	47.700 ± 1.786**
Low dose purified fraction 0.81 mg/kg	32.125 ± 0.618	31.820 ± 0.617	41.000 ± 1.048	38.780 ± 3.638**	53.700 ± 0.538	57.800 ± 3.597**
Medium- dose purified fraction 1.2 mg/kg	33.900 ± 2.050	31.666 ± 0.517	37.600 ± 2.181	32.120 ± 1.540**	55.600 ± 1.777	63.600 ± 1.435**
High-dose purified fraction 1.6 mg/kg	30.320 ± 0.226	30.520 ± 0.865	41.400 ± 1.661	41.000 ± 3.271**	53.800 ± 1.959	58.400 ± 2.785**

* Means ± standard error of the mean.

** p < 0.05.

The high-dose group (1.6 mg/kg) showed marked increases in hemoglobin and red blood cell counts compared with the untreated control and exhibited values similar to the oxfendazole group.

The lymphocyte percentage improved significantly in the medium-dose group compared with the positive control (p < 0.05). Neutrophil counts also increased notably in the treated groups, indicating enhanced immune response and recovery.

No adverse effects on hematological indices were observed, confirming the safety and biocompatibility of the purified alkaloid fractions in goats.

## DISCUSSION

### Phytochemical and pharmacological basis of *N. tabacum* activity

Plant-derived bioactive compounds have been extensively explored as safer and more sustainable alternatives to synthetic anthelmintics, which are increasingly compromised by resistance due to indiscriminate use. The wide phytochemical diversity of medicinal plants presents promising opportunities for drug discovery and veterinary applications [42].

*N. tabacum* (tobacco) contains numerous biologically active constituents, mainly alkaloids, flavonoids, and phenolic compounds, with recognized antiparasitic, antimicrobial, and antioxidant activities. These phytochemicals disrupt parasite metabolism and enhance host resilience. Nicotine, a predominant alkaloid in *N. tabacum*, acts through acetylcholine-gated ion channels, specifically nicotinic-sensitive acetylcholine receptors, which have been identified as validated but underutilized pharmacological targets in nematodes such as *H. contortus* [53].

Beyond its conventional association with tobacco toxicity, modern pharmacological research has shifted focus toward exploiting *N. tabacum* extracts for parasitic control and as potential natural pesticides [54].

### *In vitro* anthelmintic efficacy of purified alkaloid fractions

The *in vitro* assays demonstrated that the ethyl acetate fraction containing nicotine exhibited superior efficacy against *H. contortus*. In the AMT, this fraction achieved 100% worm mortality at concentrations of 3–5 mg/mL (mean: 10.00 ± 0.00), outperforming albendazole, which produced only 50% mortality at 0.25 mg/mL (mean: 5.33 ± 0.33). These findings highlight the potent nematocidal activity of *N. tabacum* alkaloids, supporting their potential as natural substitutes for conventional anthelmintics.

Similarly, in the EHA, the ethyl acetate fraction achieved complete (100%) inhibition of egg hatching at 4 and 5 mg/mL (mean: 25 ± 0 eggs), comparable to albendazole. In contrast, the petroleum ether, distilled water, and ethanol–water fractions displayed moderate inhibition (12.66 ± 0.33, 14 ± 1.5, and 13.66 ± 2.40 eggs/well, respectively). These results confirm that the alkaloid-enriched ethyl acetate fraction possesses the highest larvicidal and ovicidal potential among the tested extracts.

### *In vivo* efficacy against benzimidazole-resistant *H. contortus*

The *in vivo* trials further substantiated the strong anthelmintic effect of the ethyl acetate fraction in naturally infected goats. The high-dose group (1.6 mg/kg) demonstrated a 76.2% reduction in fecal egg counts (EPG) on day 14, exceeding the efficacy of oxfendazole (69.7%). The medium and low doses showed 62.1% and 41.9% reductions, respectively, reflecting a dose-dependent response.

These findings suggest that *N. tabacum* alkaloids, particularly nicotine, could serve as effective therapeutic agents against benzimidazole-resistant *H. contortus*. The improved efficacy over synthetic anthelmintics further underscores their potential role in managing anthelmintic resistance in small ruminant production systems.

### Safety and physiological response in treated goats

Evaluation of liver function and hematological profiles confirmed the safety of *N. tabacum* alkaloid fractions in goats. No significant differences (p > 0.05) were observed in ALT and AST levels compared with the oxfendazole control group, indicating the absence of hepatotoxicity. The highest tested dose (1.6 mg/kg) remained well within previously established non-toxic limits for livestock [55, 56]. No adverse signs, such as tremors, hypersalivation, or altered heart rate, were observed throughout the experiment.

Serum protein and CBC parameters also improved significantly (p < 0.05) across all treated groups, particularly in total protein and red blood cell indices by day 14. These physiological improvements likely reflect reduced blood loss following the paralysis and death of *H. contortus*, which feeds on the host’s blood. Consequently, the restoration of serum proteins and hemoglobin levels demonstrates both therapeutic efficacy and physiological safety of the purified fractions.

### Comparison with previous studies

The present findings align with earlier studies confirming the anthelmintic activity of alkaloids. For example, berberine and piperine inhibited egg hatching by 90%, harmaline by 80%, and berberine reduced larval motility by 98% [48]. In this study Davuluri *et al.*, [57] nicotine-based fractions achieved 100% inhibition of egg hatching and larvicidal activity, surpassing the efficacy of previously reported alkaloids.

Similar *in vitro* studies Dhadde Gurunath *et al*. [58] demonstrated that extracts from *Anacardium occidentale*, *Illicium verum*, and *Artocarpus heterophyllus* exhibited strong anthelmintic effects at 6 mg/mL, but *in vivo* validation was lacking. Our study bridges this gap by confirming both *in vitro* and *in vivo* efficacy of purified *N. tabacum* fractions. Furthermore, *Ocimum sanctum* aqueous extracts in sheep reduced EPG by 77.64% after 14 days at 5 g/animal [59], comparable to the 76.2% reduction observed in this study. Previous studies by Githiori *et al*. [60] and Gradé *et al*. [61] have reported the toxic doses of nicotine in various animal species. In the present study, the dosage was determined based on LD_50_ estimations and prior toxicity data obtained from rat models. However, unlike previous studies, our study additionally evaluated serum proteins, liver function, and CBC to confirm the absence of toxicological effects.

### Standardization and future perspectives

Despite the promising outcomes, several challenges remain in translating plant-based anthelmintics into standardized veterinary formulations. Variations in plant genotype, soil composition, climate, and extraction methods can lead to inconsistent concentrations of bioactive compounds. Establishing standardized protocols for extraction, purification, and quantification is essential to ensure reproducibility and therapeutic reliability.

Future studies should focus on:

- Conducting dose–response and toxicity profiling in multiple animal species.
- Evaluating pharmacokinetics, bioavailability, and drug disposition.
- Comparing oral and parenteral routes for optimal absorption.
- Assessing long-term efficacy and field performance across different farm environments.

Pilot-scale studies incorporating behavioral and biochemical monitoring will further refine dosage safety. Comprehensive investigations into the mechanism of action at molecular and receptor levels will also advance understanding of how *N. tabacum* alkaloids interact with nematode neurophysiology.

## CONCLUSION

This study demonstrated that purified alkaloid-rich fractions of *N. tabacum*, particularly the ethyl acetate fraction containing nicotine, possess potent anthelmintic efficacy against benzimidazole-resistant *H. contortus* in goats. The *in vitro* assays revealed complete (100%) adult worm mortality at 3–5 mg/mL and total egg hatch inhibition at 4–5 mg/mL, with an LD_50_ of 0.323 mg/mL. The *in vivo* trials supported these findings, with the high-dose group (1.6 mg/kg) achieving a 76.2% reduction in fecal egg counts, exceeding the efficacy of oxfendazole (69.7%). The ethyl acetate fraction also improved serum protein levels, hemoglobin concentration, and red blood cell count without inducing hepatic or hematological toxicity, confirming its safety and therapeutic potential in small ruminants.

The findings highlight *N. tabacum* as a promising, low-cost, and eco-friendly source of natural anthelmintic compounds. The use of purified plant-derived alkaloid fractions can reduce dependence on synthetic anthelmintics, delay the emergence of resistance, and promote sustainable livestock production. This approach directly supports the objectives of the United Nations Sustainable Development Goals, particularly SDG 2 (Zero Hunger) and SDG 3 (Good Health and Well-being) by improving animal health and productivity, SDG 12 (Responsible Consumption and Production) through sustainable resource use, and SDG 15 (Life on Land) by encouraging environmentally responsible farming practices.

The major strength of this study lies in its integrated design, combining *in vitro* and *in vivo* assessments, phytochemical characterization through chromatographic analysis (TLC and HPLC), and comprehensive hematobiochemical evaluation, thereby providing robust and reproducible evidence of efficacy and safety. However, limitations include the relatively small sample size (n = 25), single-farm study design, and lack of molecular investigation into the mechanism of action at the receptor level. These constraints may affect generalizability and warrant larger-scale and mechanistic studies to confirm and expand these findings.

Future research should focus on standardizing extraction and purification protocols, assessing pharmacokinetics and bioavailability, exploring molecular receptor interactions, and evaluating efficacy across diverse agro-ecological settings and animal species. Investigations into synergistic combinations of *N. tabacum* alkaloids with other botanicals or probiotics may further enhance their therapeutic efficacy and sustainability.

The purified alkaloid fractions of *N. tabacum* demonstrated excellent efficacy, safety, and potential as a plant-based alternative to synthetic anthelmintics. Their integration into sustainable parasite-control programs aligns with the One Health concept and contributes meaningfully to achieving SDGs 2, 3, 12, and 15, ensuring healthier livestock, safer environments, and resilient agricultural systems.

## AUTHORS’ CONTRIBUTIONS

WS, RN, and KA: Conceived the idea. WS, KH, and MSY: Designed the experiments. MSY: Performed the experiments and analyzed the data. WS and MSY: Drafted and revised the manuscript. All authors have read and approved the final version of the manuscript.
